# Increased B Cell ADAM10 in Allergic Patients and Th2 Prone Mice

**DOI:** 10.1371/journal.pone.0124331

**Published:** 2015-05-01

**Authors:** Lauren Folgosa Cooley, Rebecca K. Martin, Hannah B. Zellner, Anne-Marie Irani, Cora Uram-Tuculescu, Mohey Eldin El Shikh, Daniel H. Conrad

**Affiliations:** 1 Center for Clinical and Translational Research, Virginia Commonwealth University, Richmond, Virginia, United States of America; 2 Department of Microbiology and Immunology, Virginia Commonwealth University, Richmond, Virginia, United States of America; 3 Department of Pediatrics, Division of Allergy and Immunology, Virginia Commonwealth University, Richmond, Virginia, United States of America; 4 Department of Pathology, Division of Anatomic Pathology, Virginia Commonwealth University, Richmond, Virginia, United States of America; 5 Experimental Medicine and Rheumatology, William Harvey Research Institute, Queen Mary University of London, London, United Kingdom; Université Libre de Bruxelles, BELGIUM

## Abstract

ADAM10, as the sheddase of the low affinity IgE receptor (CD23), promotes IgE production and thus is a unique target for attenuating allergic disease. Herein, we describe that B cell levels of ADAM10, specifically, are increased in allergic patients and Th2 prone WT mouse strains (Balb/c and A/J). While T cell help augments ADAM10 expression, Balb WT B cells exhibit increased ADAM10 in the naïve state and even more dramatically increased ADAM10 after anti-CD40/IL4 stimulation compared C57 (Th1 prone) WT B cells. Furthermore, ADAM17 and TNF are reduced in allergic patients and Th2 prone mouse strains (Balb/c and A/J) compared to Th1 prone controls. To further understand this regulation, ADAM17 and TNF were studied in C57Bl/6 and Balb/c mice deficient in ADAM10. C57-ADAM10^B-/-^ were more adept at increasing ADAM17 levels and thus TNF cleavage resulting in excess follicular TNF levels and abnormal secondary lymphoid tissue architecture not noted in Balb-ADAM10^B-/-^. Moreover, the level of B cell ADAM10 as well as Th context is critical for determining IgE production potential. Using a murine house dust mite airway hypersensitivity model, we describe that high B cell ADAM10 level in a Th2 context (Balb/c WT) is optimal for disease induction including bronchoconstriction, goblet cell metaplasia, mucus, inflammatory cellular infiltration, and IgE production. Balb/c mice deficient in B cell ADAM10 have attenuated lung and airway symptoms compared to Balb WT and are actually most similar to C57 WT (Th1 prone). C57-ADAM10^B-/-^ have even further reduced symptomology. Taken together, it is critical to consider both innate B cell levels of ADAM10 and ADAM17 as well as Th context when determining host susceptibility to allergic disease. High B cell ADAM10 and low ADAM17 levels would help diagnostically in predicting Th2 disease susceptibility; and, we provide support for the use ADAM10 inhibitors in treating Th2 disease.

## Introduction

A disintegrin and metalloproteinases (ADAMs) are zinc dependent proteinases, which perform ectodomain cleavage of transmembrane proteins. ADAM10 and ADAM17, or tumor necrosis factor alpha (TNF) converting enzyme (TACE), are structurally related and share overlapping substrates including TNF [1,2]. ADAM10 contributes to allergic disease being the principal sheddase of CD23, the low affinity IgE receptor, which promotes IgE production [3,4] and is increased in allergic patients’ sera [5]. In an experimental asthma model [4,6], ADAM10 inhibitor administration significantly attenuated airway hyperreactivity, suggesting that increased ADAM10 activity predisposes to allergic disease.

The Th1/Th2 paradigm is attributed to differences in CD4+ T cell response and has been studied extensively in both mice and humans. Allergic diseases are skewed towards a Th2 phenotype and classic Th1 (such as C57Bl/6 and SJL/J) and Th2-prone (such as Balb/c and A/J) strains were characterized as high (Balb/c, A/J), intermediate (C57Bl/6), and low (SJL/J) IgE responders based on *in vivo* IgE production post immunization [7]. Whether B cells from Th1 or Th2-biased mouse strains have intrinsic differences in ADAM10 and ADAM17 and if such differences affect IgE production has never been elucidated.

In the absence of B cell ADAM10 (B-ADAM10) in C57Bl/6 mice (C57-ADAM10^B-/-^), a key compensatory increase in ADAM17 and thus TNF shedding [8] results in aberrant B cell/T cell localization, reduced germinal center formation, decreased follicular dendritic cell (FDC) maturation, excessive collagen deposition, and increased high endothelial venule (HEV) formation [8–11]. Furthermore, C57-ADAM10^B-/-^ mice were less susceptible to airway hypersensitivity induction, suggesting a specific role for B-ADAM10 in provoking allergic disease [6]. This phenotype is C57-ADAM10^B-/-^ mice is not found, however, in Balb-ADAM10^B-/-^ mice thus posing a critical question about ADAM10 and ADAM17 regulation in different Th contexts. Herein, we explore key differences between typical Th1 and Th2 prone strains with respect to ADAM10, ADAM17, and TNF and in ADAM17 regulation following ADAM10 deletion.

We further extend our studies to allergic disease situations both in humans using active allergic rhinitis patients and in mice by using a clinically relevant house dust-mite (HDM) airway model. We investigate specifically whether intrinsic differences in B cell ADAM10 levels, independent of Th bias, regulates allergy induction and severity and whether this regulation is associated with modulation of B cell ADAM17 and TNF and associated changes in secondary lymphoid follicular architecture.

## Materials and Methods

### Ethics Statement

All human studies were approved by the Virginia Commonwealth University IRB. Patients were informed of the study and consented by Dr. Anne-Marie Irani using the approved IRB survey: IRB #00870. Patients were described the IRB approved survey and then signed the waiver enclosed. All animal care and experimental protocols were approved by Virginia Commonwealth University Institutional Animal Care and Use Committee and were in accordance with NIH guidelines. Mice were sacrificed by isoflurane inhalation followed by cervical dislocation. Anesthesia used for intranasal injections was mixed oxygen/isoflurane.

### Mice

C57Bl/6 ADAM10^B-/-^ (CD19-cre^+^) mice (C57-ADAM10^B-/-^) were generated [12] and backcrossed to Balb/c (Balb-ADAM10^B-/-^) for 8 generations and compared to littermate controls (CD19-cre^-^). A/J, SJL/J, C57Bl/6, and Balb/c WT were from Jackson Laboratories. All mice were 6–12 weeks when used. All animal care and experimental protocols were approved by Virginia Commonwealth University Institutional Animal Care and Use Committee and were in accordance with NIH guidelines.

### Human studies

All human studies were approved by the Virginia Commonwealth University IRB. Sixteen symptomatic allergic rhinitis patients and 16 controls were recruited for the study by Dr. Anne-Marie Irani at VCU. Inclusion criteria included active allergic rhinitis symptoms and a documented positive skin test or ImmunoCAP to an antigen as consented in IRB #00870 (S1 Fig Peripheral blood mononuclear cells (PBMC) were isolated from peripheral blood using Ficoll, stained, and remaining PBMC underwent CD19 B cell selection using magnetic beads (Miltenyi Biotec). B cells were cultured for 2 days (or 5 days for CD23 ELISA) with 200 ng/mL human IL-21 (ATCC), 1 μg/mL anti-CD40 (Clone G28-5, ATCC), and 10 ng/mL rhIL-4 (R&D). Stimulated B cells were harvested, stained for flow cytometry, analyzed by qPCR, and sCD23 determined by ELISA [13]. HDM *Dermatophagoides pteronyssinus* IgE was determined by ImmunoCAP (Phadia, 14-4107-01).

### Murine B cell culture and TNF ELISA

Positively selected B220+ splenic B cells (Miltenyi Biotec) were grown for 1–3 days in cultures containing 1000 units IL-4 and 50 μg/mL LPS (*E*. *coli* 0111:B4, Sigma) or 1.25μg/mL purified anti-mouse CD40 (Biolegend) [8]. Supernatants were analyzed for sTNF by ELISA (eBioscience).

### Murine HDM studies

C57Bl/6 WT, C57-ADAM10^B-/-^, Balb/c WT, and Balb-ADAM10^B-/-^ mice were intranasally exposed to 25μL saline or 15μg/25μL HDM extract (Greer Laboratories) (S2 Fig) [14]. Bronchoconstriction was assessed using Flexivent (Scireq, Montreal, QC, Canada) as previously described [15]. Airway resistance (Rrs) was determined at increasing doses of methacholine (5, 10, 25 mg/ml) and presented as percent increase from PBS baseline. HDM specific IgE determined by ELISA as described [16]. Briefly, plates were coated with 20μg/mL HDM extract in 50mM carbonate buffer, blocked with SuperBlock (Thermo Scientific), detected using goat anti-mouse IgE (Abcam), followed by addition of Streptavidin-HRP (Southern Biotechnologies), and color developed with tetramethylbenzidine + substrate (BD Biosciences). Reaction was stopped with 1N H_2_SO_4_ and absorbance read at 450nm.

BALF cells were stained with APC-B220, APC-CD3, BV421-1A/1E, BV605-CD11c, and PE-CCR3 (Biolegend) after FcR blockade with anti-mouse CD16/32 (2.4G2). Samples were examined on a BD Fortessa and analyzed with FCS Express, v. 4. using the gating strategy described [17]. BALF supernatants were analyzed for MUC5AC protein by ELISA as described [15]. Briefly, BALF was diluted and 75μL was incubated with 75μL carbonate buffer. Samples were incubated overnight without a lid at 37°C then washed three times with PBS. Plates were blocked, detected with anti-Muc5AC mouse monoclonal antibody (Pierce, 1:100), followed by addition of goat anti-mouse IgG-HRP (Southern Biotechnologies,1:10,000), developed with tetramethylbenzidine + substrate (BD Biosciences), stopped with 1N H_2_SO_4_, and absorbance read at 450nm.

### Murine Lung histology

Five μm sections of formalin-fixed, paraffin-embedded murine lung tissue were stained with hematoxylin and eosin (H&E) and Periodic acid-Schiff (PAS) (AML Laboratories, Inc. Baltimore, MD) and photographed using Olympus-DP70 camera on Olympus-BX41 microscope. Histopathologic evaluation of H&E stained lung sections from at least 4 mice per group was performed by a pathologist in a blinded fashion using a semi-quantitative scoring system on a Nikon Eclipse microscope. Peribronchiolar and perivascular inflammatory cellular infiltration were scored separately as follows: 0, no or occasional cells; 1, few or loosely arranged cells; 2, focal involvement of lung parenchyma with majority of airways or vessels having rings (partial or complete) of inflammatory cells one cell layer deep; 3, patchy involvement of lung parenchyma with majority of airways or vessels having rings (partial or complete) of inflammatory cells two to four cell layers deep; 4, extensive involvement of lung parenchyma with majority of airways or vessels having rings (partial or complete) of inflammatory cells more than four cell layers deep. Individual scores for peribronchiolar and perivascular inflammation were added together for a total maximum score of 8.

### qPCR and western blotting

Total RNA was extracted using TRIzol reagent (Invitrogen) and quantified by NanoDrop-100 spectrophotometer. Real-time quantitative PCR was performed with an iQ5 (Bio-Rad Laboratories) [8]. TaqMan quantitative PCR assays (Applied Biosystems, Life Technologies) were run using primers/probes: TNF (Mm00443258 or Hs01113624), ADAM10 (Mm00545742 or Hs00153853), ADAM17 (Mm00456428 or Hs01041915), 18s (Mm03928990), and GAPDH (Hs02758991). Fold variation analyzed using delta-delta Ct (∆∆Ct) formula [18].

Proteins were assessed by western blot [8] using rabbit-anti-β-actin peroxidase (Sigma-Aldrich) or anti-ADAM17 (Abcam) followed by HRP-anti-rabbit IgG and detected with SuperSignal West Pico Chemiluminescent Substrate (Thermo Scientific).

### Flow cytometry

Mouse B220+ B cells or human PBMC were blocked with 10 μg 2.4G2 or human FcR blocker (Miltenyi Biotec), respectively. Human PBMC was stained with anti-human PE-CD19, APC-CD14, or PE-CD3 (Biolegend). Mouse and human cells were washed, fixed, permeabilized, blocked again, and stained with their respective antibodies: FITC-hADAM10 (R&D), PE-mADAM10 (R&D) and/or rabbit-anti-ADAM17 (Abcam) followed by DyLight-649 anti-rabbit IgG (Biolegend). Isotype control for hADAM10 is mouse IgG2b FITC (R&D), for mADAM10 is Rat IgG2a PE (R&D), and for ADAM17 is rabbit IgG. Tyramide Signal Amplification (Kit #26) was used for mouse B cell TNF staining as described [8]. Dead cells and aggregates were excluded by scatter gating, examined on a BD Canto, and analyzed with FCS Express, v.4.

### Immunohistochemistry and confocal microscopy

Ten μm frozen mouse LN sections were dual and triple-labeled for FDCs (PE-anti-CD21/CD35, Biolegend, 123410), B cells (Alexa-Fluor-647-anti-CD45R/B220, Santa Cruz Biotechnology, sc-19597- Alexa-Fluor-647), HEVs (anti-peripheral node addressin (pNAD), Biolegend, 120804), T cells (Anti-CD90.2/Thy-1.2-PE, Southern Biotech, 1750-09L), collagen-1 (Abcam, ab21286), and TNF (Abcam, ab34674). Instrumentation and quantitative assessment of sections performed as previously described [8]. TNF labeling was enhanced using Fluorescein-TSA (TSA Plus Fluorescein System, Perkin Elmer, NEL741001KT) [8].

### Statistical analysis

**Fig 1:**
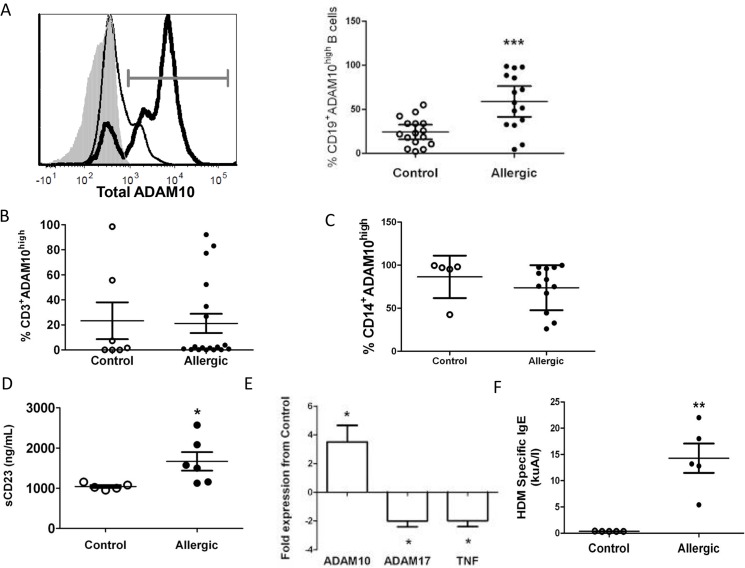
Allergic patient B cells exhibit increased ADAM10 and sCD23 but decreased ADAM17 and TNF. (A) Total ADAM10 in naïve CD19^+^ B cells from control (thin line, open dot) and allergic (bold line, black dot) patients; dot plot (right) shows percent of total B cells staining high in ADAM10 (grey gate). Isotype control is shaded grey in histogram. (B,C) Total ADAM10 on naïve T cells (B) and monocytes (C). (D) sCD23 from control (open dot) or allergic (black dot) supernatants. (E) Naïve B cells from 4 allergic and 4 control patients analyzed for ADAM10, ADAM17, and TNF message normalized to GAPDH. Significance (*) indicates ≥ 2 fold change between allergic and control B cells for respective gene. (F) HDM specific IgE levels in sera of control (open dot) or allergic (black dot) patients determined by ImmunoCAP. <0.35kuA/l considered a negative result. *p<0.05, **p<0.005, ***p<0.0005.

**Fig 2:**
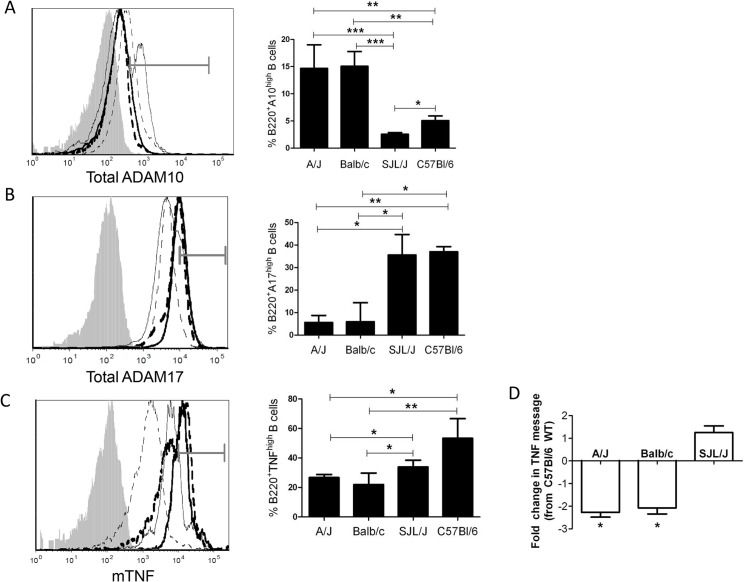
Increased B-ADAM10 and decreased ADAM17 and TNF in Th2 biased strains. Naïve B220^+^ B cells (A, B) stained for total ADAM10 (A) or ADAM17 (B) expression by flow cytometry. (C) TNF coexpression on 3 day stimulated (anti-CD40+IL4) B220^+^ B cells by TSA. (A,B,C) Bar graph represents percent total B cells staining high in ADAM10 (A), ADAM17 (B), or TNF (C). (A,B,C) Balb/c (──)A/J (——), C57Bl/6 (▬▬), SJL (▪▪▪▪▪), respective isotype control (shaded grey) in representative overlay (left); grey gate demonstrates high stained population. n = 8 per group, 3 independent studies. *p<0.05, **p<0.005, ***p<0.0005. (D) TNF message from 3 day stimulated (anti-CD40 + IL4) B220^+^ B cells. Presented as fold change from C57Bl/6 WT. * signifies ≥2 fold change between C57Bl/6 WT and the listed WT strain.

**Fig 3:**
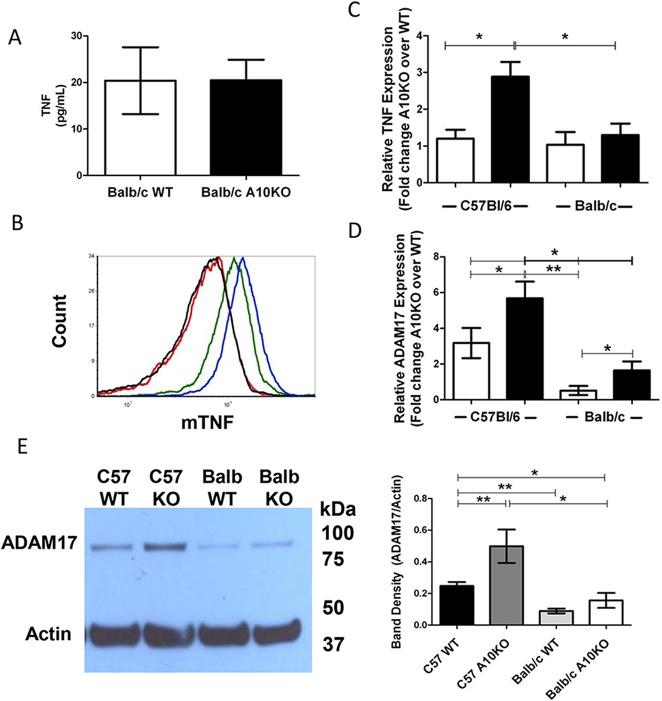
B cell TNF and ADAM17 regulation in C57Bl/6 and Balb/c ADAM10^B-/-^ (A10K0) and WT. (A) sTNF or (B) mTNF from 3 day stimulated (LPS + IL4) B cell cultures; (B) Balb/c WT (black), Balb/c A10KO (red), C57Bl/6 WT (green), and C57Bl/6 A10KO (blue) B cells. (C) Fold change (A10KO over WT) in relative TNF message from naïve (white) or 3 day stimulated (anti-CD40+IL4) (black) B cells normalized to 18s. (D) Fold change (A10KO over WT) in relative ADAM17 expression normalized to 18s for naïve (white) or 3 day stimulated (black) B cells. (C,D) * signifies ≥2 fold change between groups. (E) ADAM17 (~93kDa) and actin (~42kDa) from 5 day stimulated (anti-CD40 + IL4) B cells (left) and band densitometry (right). KO = A10KO. n = 9 per group, 3 independent studies. *p<0.05, **p<0.005.

**Fig 4:**
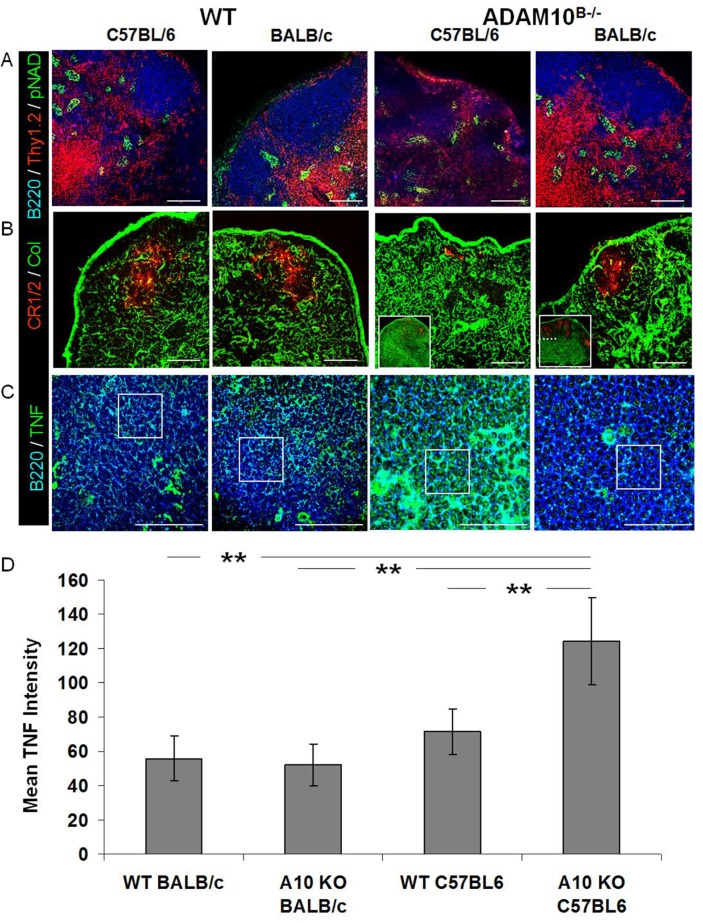
Balb-ADAM10^B-/-^ LN exhibit WT architecture unlike C57-ADAM10^B-/-^ nodes. Naïve LN sections from C57Bl/6 and Balb/c WT and ADAM10^B-/-^; (A) B cell (blue, B220), T cell (red, Thy1.2), and HEV (green, pNAD); (B) FDC reticula (red,CR1/2), collagen (green), and cortico-medullary junction (dotted line in inset box); (C) TNF staining (green), B cell (blue, B220) follicle. (D) Average TNF staining intensity representing 12 follicle sections per group. Scale bar, 50μm. **p<0.005.

**Fig 5:**
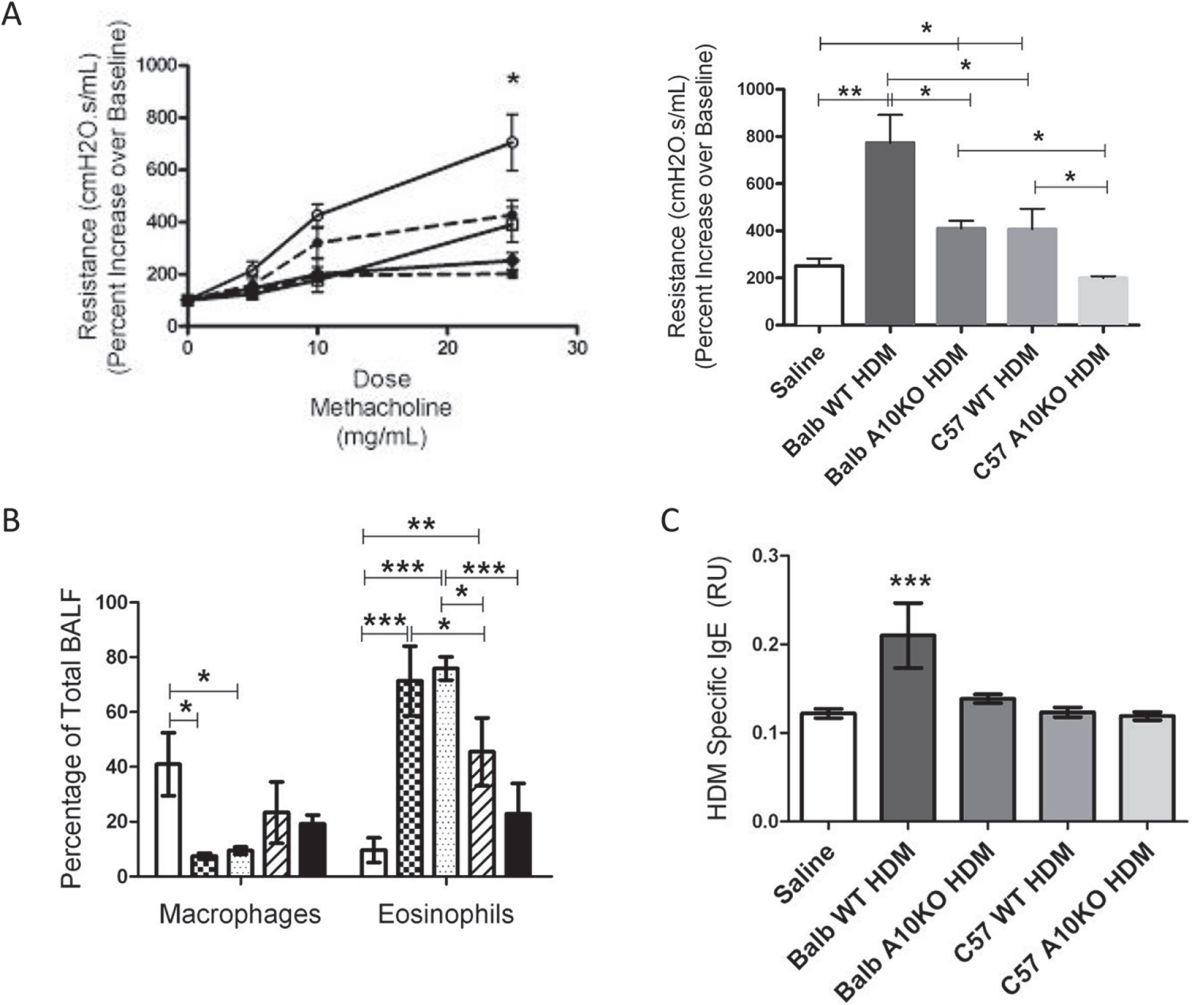
B-ADAM10 deletion attenuates bronchoconstriction and HDM specific IgE. (A) Airway resistance (cmH20.s/mL) with increasing doses of methacholine (left); Balb-WT (──,○), Balb-ADAM10^B-/-^(——,●), C57-WT (──,□), C57-ADAM10^B-/-^ (——,○), Saline (──,●) presented as fold increase from saline control. Bar graph (right) represents 25 mg/mL methacholine dose. (B) Percent macrophages (left) and eosinophils (right) from total BALF determined by flow cytometry; Saline control (white), Balb WT (checkered), Balb-ADAM10^B-/-^ (dotted), C57 WT (slash), and C57-ADAM10^B-/-^ (black). (C) HDM specific IgE production. n = 7–9 per group, 3 independent experiments. All mice immunized with saline demonstrated comparable results. For simplicity, saline represents Balb WT mice given saline. *p<0.05, **p<0.005, ***p<0.005.

**Fig 6:**
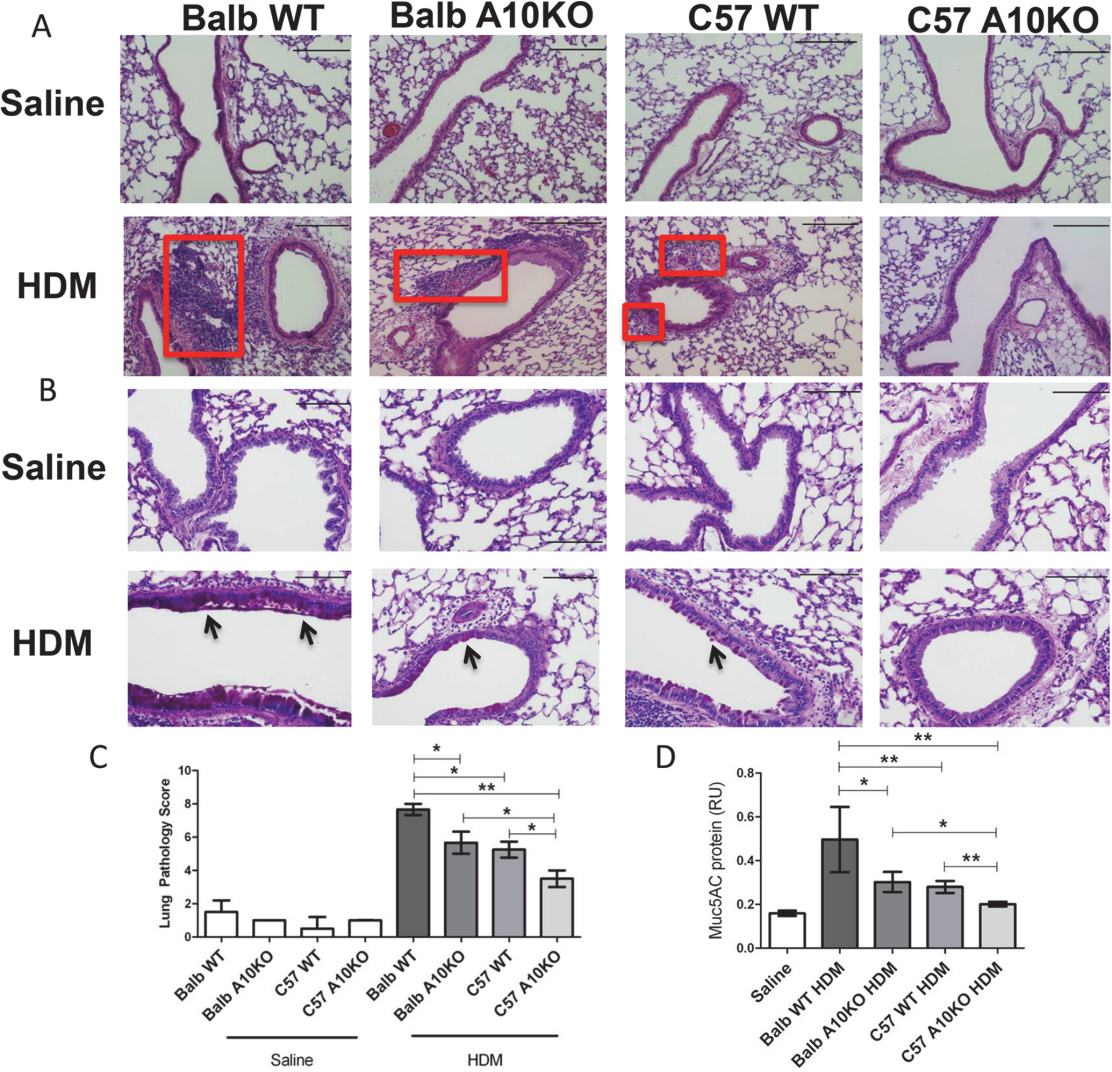
B-ADAM10 deletion reduces cellular infiltration, goblet cell metaplasia, and mucus production in a strain dependent manner. (A) H&E stain for lung tissue demonstrates peribronchiolar and perivascular inflammatory cellular infiltration (red box); 10x magnification, scale bar 200 μm. (B) Goblet cell metaplasia determined by PAS stain; note dark pink mucin producing cells (black arrow) and alveolar epithelium thickness; 20x magnification, scale bar 100 μm. (C) Lung pathology score representing quantitation of peribronchiolar and perivascular inflammatory cellular infiltration on H&E stained lung sections; 2 sections per mouse and at least 4 mice per group were assessed. (D) MUC5AC protein. n = 7–9 per group, 3 independent experiments. All mice immunized with saline demonstrated comparable results. For simplicity, saline represents Balb WT mice given saline. *p<0.05, **p<0.005.

Normal distribution of data sets was determined using the Shapiro-Wilk normality test with SigmaPlot 12.5. Furthermore, the mean and medians were close for data sets indicative of normal distribution. Significance (p<0.05) was calculated using unpaired two-tailed Student *t* tests (Figs 1–5) and one-way ANOVA with Tukey post-test (Figs 5 and 6) in GraphPad Prism. Error bars represent SD; except in Fig 1A where error bars represent 95% confidence interval.

## Results

### Allergic patients exhibit increased B cell ADAM10, CD23, and IgE

Given the increased circulating sCD23 in the sera of allergic patients [5], we hypothesized that actively allergic patients would exhibit increased B-ADAM10 compared to controls. Naïve B cells from 16 allergic patients exhibited significantly increased B-ADAM10 compared to 16 controls (Fig 1A) by flow cytometric analysis. Contrastingly, T cells (Fig 1B) and monocytes (Fig 1C) from allergic and control patients revealed comparable ADAM10 expression. Thus, only B-ADAM10 expression served as an allergy-associated differential indicator. Furthermore, increased sCD23 in allergic B cell cultures indicated high ADAM10 activity in allergic B cells (Fig 1D). B-ADAM10 message in allergic patients was increased nearly 5-fold, while ADAM17 and TNF message were decreased 2-fold compared to controls (Fig 1E).

Given that increased B-ADAM10 leads to increased IgE production through CD23 cleavage [4], we next compared serum antigen-specific IgE between allergic and control patients. We were reliant on each patient self-reporting a specific antigen to which he/she was allergic in order to perform an antigen specific ImmunoCAP [19]. Five of 16 allergic patients self reported HDM sensitivity and ImmunoCAP demonstrated positive HDM specific IgE levels (>0.35 kuA/I) (Fig 1F). Five control patients, who are also quite likely exposed to HDM daily but do not develop allergy, demonstrated negative ImmunoCAP results (<0.35 kuA/I) or very low levels (0.41kuA/l) (Fig 1F). We next compared each individual patient’s HDM-specific IgE result to his/her respective B cell ADAM10 expression (Table 1). In general, all control patients exhibited very low or negative HDM specific IgE and low ADAM10 expression (< 25%) (Table 1). As HDM specific IgE levels increased, B cell ADAM10 expression also increased in allergic patients. One allergic patient, however, had a lower HDM specific IgE result at 5.4 kuA/l, but demonstrated a high B cell ADAM10 level (69%) (Table 1). This patient, specifically, reported multiple antigens to which he/she is allergic. Therefore, 5.4 kuA/l reflects only the level of HDM specific IgE and is most likely lower than the total antigen specific IgE present in the serum. Overall, Fig 1 and Table 1 demonstrates a correlation between increased serum antigen-specific IgE and increased B cell ADAM10 in allergic patients.

**Table 1:** Increased HDM specific IgE correlates with increased B cell ADAM10 expression.

Control		Allergic	
HDM Specific IgE (kuA/l)	Percent CD19^+^ADAM10^high^	HDM Specific IgE (kuA/l)	Percent CD19^+^ADAM10^high^
<0.35	13	5.4	69
<0.35	18	12.8	48
<0.35	22	15.0	51
<0.35	25	18.0	60
0.41	15	22.0	72

Direct comparison of individual patient’s HDM specific IgE determined by ImmunoCAP and B cell ADAM10 expression determined by flow cytometry.

### Increased B-ADAM10 and decreased ADAM17 and TNF in Th2 prone strains

Since B cell levels of ADAM10, ADAM17, and TNF mRNA expression were clearly different in control and allergic patients; and allergy is a Th2-dominated disorder; we sought to confirm our results in classic Th1 and Th2 prone mouse strains. Naïve B cells from Th2 prone Balb/c and A/J expressed higher total ADAM10 (Fig 2A) and decreased total ADAM17 (Fig 2B) compared to Th1 prone C57Bl/6 and SJL/J. Upon stimulation *in vitro*, B cells from the chosen Th1 prone strains expressed increased mTNF compared to the chosen Th2 prone strains (Fig 2C). Furthermore, TNF message was lower in Th2 prone A/J and Balb/c mice compared to C57Bl/6 WT by ≥2 fold (Fig 2D). C57Bl/6 WT TNF expression was not significantly different from SJL/J levels of TNF expression (Fig 2D). B cell ADAM10 in both Th1 prone-C57Bl/6 and Th2 prone-Balb/c strains increased following *in vitro* stimulation (S1 Table). However, Th2 prone Balb/c maintained a consistently higher level of B cell ADAM10 even after equal stimulation (S1 Table). This finding demonstrates that T cell help augments ADAM10 expression but does not provide the mechanism by which B-ADAM10 is initially increased in Th2 prone over Th1 prone strains.

### Differential ADAM17 and TNF in Balb-ADAM10^B-/-^ compared to C57- ADAM10^B-/-^

C57-ADAM10^B-/-^ B cells exhibit a compensatory increase in ADAM17 expression and function resulting in excessive TNF cleavage, which provided the mechanism for the aberrant secondary lymphoid tissue architecture noted in these mice [8,9]. Given our results in Figs 1 and 2, we predicted that Th2 prone Balb-ADAM10^B-/-^ B cells would exhibit reduced ADAM17 and TNF compared to Th1 prone C57-ADAM10^B-/-^ B cells, which may help to maintain WT lymphoid tissue architecture. Distinctively, sTNF production by Balb-WT and Balb-ADAM-10^B-/-^ B cells was comparable following *in vitro* stimulation (Fig 3A) unlike our previous report in C57Bl/6 [8], which demonstrated significantly increased sTNF production by C57-ADAM10^B-/-^ B cells compared to WT in the same *in vitro* conditions. Furthermore, mTNF in stimulated B cells is comparable between Balb-WT and Balb-ADAM10^B-/-^ but reduced compared to C57-WT and even further reduced compared to C57-ADAM10^B-/-^ (Fig 3B). Similarly to C57, Balb-WT and Balb-ADAM10^B-/-^ express comparable TNF message in the naïve state (Fig 3C). Upon stimulation, however, Balb-ADAM10^B-/-^ B cells fail to significantly increase TNF message above Balb-WT levels unlike C57-ADAM10^B-/-^ B cells (Fig 3C). Furthermore, C57-ADAM10^B-/-^ B cells exhibit increased TNF message compared to Balb-ADAM10^B-/-^ (Fig 3C).

Given that TNF levels were reduced in Balb-ADAM10^B-/-^ compared to C57-ADAM10^B-/-^ B cells, we next compared the levels of ADAM17 protein and message. Unlike C57Bl/6, ADAM17 message in naïve Balb-WT and Balb-ADAM10^B-/-^ B cells was comparable and increased to 2-fold higher expression in Balb-ADAM10^B-/-^ B cells upon stimulation (Fig 3D). C57-ADAM10^B-/-^ B cells exhibited increased ADAM17 protein (Fig 3E) compared to C57-WT, but also compared to Balb-WT and most interestingly, Balb-ADAM10^B-/-^. Furthermore, ADAM17 protein was comparable between Balb-WT and Balb-ADAM10^B-/-^, which is consistent with TNF production in Fig 3A (Fig 3E)

### Unlike C57-ADAM10^B-/-^, Balb-ADAM10^B-/-^ exhibit normal secondary lymphoid tissue architecture

Given the marginal changes in TNF and ADAM17 (Fig 3) in Balb-ADAM10^B-/-^ compared to our previous report in C57-ADAM10^B-/-^ [8], we hypothesized Balb-ADAM10^B-/-^ lymph nodes would maintain WT architecture. Indeed, Balb-ADAM10^B-/-^ exhibited comparable primary follicular characteristics to C57-WT and Balb-WT, including normal B cell/T cell compartmentalization (note the lack of B/T compartmentalization in the C57-ADAM10^B-/-^ compared to other groups) (Fig 4A), number and size of HEVs (Fig 4A), FDC reticular development (note minimal CD21^+^ FDC in C57-ADAM10^B-/-^) (Fig 4B, inset Fig 4B), and collagen deposition (note collagen is normally sparse but is increased in C57-ADAM10^B-/-^) (Fig 4B). Furthermore, TNF labeling (boxed regions of Fig 4C) demonstrated that C57-ADAM10^B-/-^ follicles displayed the highest TNF intensity compared to C57-WT, Balb-WT, and Balb-ADAM10^B-/-^ (Fig 4C), with the latter groups all having comparable TNF staining (Fig 4C), which is quantified in Fig 4D. These results provide further support to the mechanism underlying the aberrant lymphoid tissue architecture in C57-ADAM10^B-/-^ mice and demonstrate the inherent difference in B cell ADAM10 and ADAM17 regulation in Th1 vs. Th2 prone mice.

### High B-ADAM10 level in the context of a Th2 environment allows optimal induction of allergic airway disease symptoms

C57Bl/6 WT exhibit a decreased response to mouse lung inflammation models compared to Balb/c including airway bronchoconstriction and IgE production, however, these findings have never been linked to B-ADAM10 levels, specifically [8,20,21]. Our results thus far indicate that Th2 prone mice and humans, whose B cells are known to produce excess IgE, exhibit increased B cell ADAM10 even in a naïve, non-stimulated state. After stimulation with anti-CD40/IL4, B-ADAM10 levels increased more dramatically in Th2 prone strain B cells as well. Therefore, we next sought to compare the consequences of B cell ADAM10 level in the context of a Th1 or Th2 immune-environment on airway hypersensitivity induction following HDM challenge, specifically with regards to IgE production. The groups considered included: B cell ADAM10 high, Th2 prone (Balb/c WT); B cell ADAM10 low, Th1 prone (C57 WT); B cell ADAM10 deleted, Th2 prone (Balb-ADAM10^B-/-^); and B cell ADAM10 deleted, Th1 prone (C57-ADAM10^B-/-^). When each group was treated intranasally with saline, all disease parameters were similar (not statistically different). For clarity, saline treated Balb WT was chosen as the representative saline group in Figs 5 and 6.

Th2 prone, B-ADAM10 high Balb WT exhibit the most severe induction of lung inflammation following HDM challenge, including increased bronchoconstriction (Fig 5A) and extensive peribronchiolar and perivascular inflammatory cellular infiltration in lung tissue (boxed regions in Fig 6A and 6C). Similarly, Balb WT exhibit the highest goblet cell metaplasia as indicated by the intense pink staining mucin (arrows in Fig 6B) and mucus (MUC5AC protein) production (Fig 6D). Furthermore, the thickness of the alveolar epithelium (Fig 6B) is greatest in the Balb/c compared to other groups with the C57-ADAM10^B-/-^ being reflective of saline controls. In accordance with our main hypothesis that increased B cell ADAM10 lends to increased IgE production, Balb WT exhibited the highest HDM-specific IgE (Fig 5C) as well. When ADAM10 is deleted from B cells but remains in the context of a Th2 environment in the Balb-ADAM10^B-/-^, bronchoconstriction (Fig 5A), goblet cell hyperplasia (Fig 6B), and mucus production (Fig 6D) are reduced significantly from Balb WT and are quite similar to the Th1 prone, B-ADAM10 low C57-WT. Furthermore, HDM-specific IgE (Fig 5C) is reduced from Balb WT levels demonstrating the critical importance of B cell ADAM10 for IgE production even in a Th2 prone environment. Lastly, B-ADAM10 deletion in the context of a Th1 environment (C57-ADAM10^B-/-^) provides the least suitable context for allergic airway disease induction. Thus, as indicated previously, C57-ADAM10^B-/-^ mice exhibited bronchoconstriction (Fig 5A), cellular infiltration of lung tissue (Fig 6A and 6C), goblet cell metaplasia (Fig 6B), and mucus production (Fig 6D) similar to saline control. While the HDM-specific IgE ELISA was not sensitive enough to detect differences between saline, C57-WT, and C57 and Balb-ADAM10^B-/-^, it does indicate the importance of both ADAM10 and strain background in IgE production as both the Balb-ADAM10^B-/-^ and C57-WT demonstrated significantly reduced HDM-specific IgE production compared to Balb WT (Fig 5C). Furthermore, B-ADAM10 deletion from Balb or C57 fails to reduce eosinophilic recruitment in BALF, but it appears to be a strain dependent phenomena as Balb/c mice regardless of B cell ADAM10 presence had increased eosinophils compared to C57 WT and C57-ADAM10^B-/-^ (Fig 5B).

## Discussion

Compelling evidence, herein, demonstrates that B-ADAM10, ADAM17, and TNF are differentially regulated in Th1 and Th2-dominated immune responses and directly influence host susceptibility to allergy and IgE production. We demonstrate that Th2 prone mouse strains (Fig 2) and humans (Fig 1) exhibit increased B cell ADAM10 and IgE (Fig 1F, Table 1 and Fig 5C), while concomitantly having reduced ADAM17 and TNF (Fig 3). With regards to mouse strains, we compared to C57-ADAM10^B-/-^ B cells to Balb-ADAM10^B-/-^ and found that Th environment is critical for ADAM17 and TNF regulation as Balb-ADAM10^B-/-^ are less adept at increasing ADAM17 and thus TNF cleavage following ADAM10 deletion (Figs 3 and 4) [8]. WT levels of TNF production by Balb-ADAM10^B-/-^ B cells allows for maintenance of proper secondary lymphoid tissue architecture and provided further evidence to the mechanism underlying abnormal architecture characteristics in C57-ADAM10^B-/-^ nodes (Fig 4) [8].

A critical question is whether B cell ADAM10 levels is strictly determined by T cell help or is an inherent characteristic of B cells from Th1 or Th2 prone strains. The answer is both. T cell help as demonstrated by *in vitro* culture with equal concentrations of anti-CD40/IL4 shows that B cell ADAM10 increases in both C57 and Balb WT B cells following stimulation (S1 Table and Fig 2). Thus, T cell help augments ADAM10 expression. Importantly, however, Balb WT B cells exhibit increased ADAM10 in the naïve state and even more dramatically increased ADAM10 after anti-CD40/IL4 stimulation compared C57-WT B cells (S1 Table). This finding indicates an innate difference between their B cells that affects their sensitivity to T cell mediated ADAM10 induction.

Next, regulation of ADAM17 and its ligand, TNF, in different Th contexts was analyzed. C57-WT B cells demonstrated increased ADAM17 in naïve B cells and increased TNF expression following *in vitro* stimulation compared to Balb-WT B cells (Fig 2). These B cell differences in the classic Th1 and Th2-biased strains extended other strains of mice as well. For our studies, we used to SJL/J and C57Bl/6 as our Th1 prone strains and A/J and Balb/c as our Th2 prone strains (Fig 2). Furthermore, Th context is critical for understanding the difference in ADAM17 and TNF regulation following B cell ADAM10 deletion as shown in Fig 3, as Balb-ADAM10^B-/-^ B cells fail to increase ADAM17 and TNF production, thus permitting WT architecture maintenance (Fig 4). Our evidence suggests that regulation of B-ADAM10 and ADAM17 is both determined by Th context (e.g. T cell help) but also on inherent differences in B cell responsiveness.

In agreement with the clinically relevant HDM model used herein, OVA-induced airway hypersensitivity was also reduced in C57-ADAM10^B-/-^ [6]. In this study using a HDM model, we directly compare C57 and Balb ADAM10^B-/-^ mice and both experienced significantly less bronchoconstriction, goblet cell metaplasia, mucus, and HDM-specific IgE production (in Balb/c) compared to their respective WT strain (Figs 5 and 6). Importantly, however, Balb- ADAM10^B-/-^ mice had lung inflammation parameters comparable to WT-C57 mice, while C57-ADAM10^B-/-^ had the lowest disease induction. Furthermore, while clinically relevant features of human asthma including antigen-specific IgE, mucus, and airway resistance were clearly ADAM10 dependent, eosinophil infiltration was strain dependent only (Fig 5). This finding does not detract from the potential use of ADAM10 inhibitor therapy, however, because blockade of eosinophil infiltration alone fails to reduce asthma related symptoms in most allergic patients [22,23]. Overall, the data indicates that B-ADAM10 plays an important role in this Th2 disease model with the highest ADAM10 expression indicating worst symptomology. While the mechanism by which ADAM10 levels influence Th2 disease is unknown, a candidate mechanism could be that ADAM10-mediated CD23 shedding increases IgE production, thus enhancing IgE cross-linking and mast cell degranulation [4]. If further validated in a larger human cohort study, ADAM10 level could serve as a potential indicator of the directionality or strength of a Th1 or Th2 response, with high ADAM10 level indicating a propensity toward enhanced Th2 responsiveness with excess IgE production.

Our data in mouse strains was validated by our findings in allergic patients (Th2 prone) who demonstrated increased B cell ADAM10 and reduced B cell ADAM17 and TNF production (Fig 1). Together this suggests that B cell-ADAM10 screening could effectively stratify allergic from non-allergic patients potentially for their susceptibility to more severe disease. An appropriate, cost-effective means to perform this task is used herein: flow cytometric analysis of ADAM10 levels on peripheral blood CD19+ B cells obtained from a minimally invasive venous puncture. Furthermore, while ADAM10 screening could be a critical diagnostic tool, ADAM10 inhibition therapy also stands to be the only mechanism by which to inhibit the initial step in the allergic cascade: IgE production. We demonstrate, herein, that B cell ADAM10 deletion reduces antigen-specific IgE production (Fig 5C). Consequently, ADAM10 inhibitors have the potential to provide anti-allergic prophylaxis when locally administered particularly to atopic patients with high B-ADAM10.

Allergic patients with increased B-ADAM10 concomitantly had reduced B cell ADAM17 and TNF, which could also provide mechanistic insight into their enhanced IgE production. Increased B cell TNF production, as seen in aging B cells, limits antibody production, suggesting a potentially protective role of ADAM17 and TNF from induction of an allergic phenotype [24]. Our findings, overall, suggest that allergy-prone B cells display an ADAM10^hi^/ADAM17^lo^/TNF^lo^ phenotype, with greater risk of atopic manifestations in Th2-biased environments. Our results are an advance in elucidating the key role of ADAM10 in allergy pathogenesis, and provide a novel approach to the diagnosis, prognosis, and treatment of atopic disorders.

## Supporting Information

S1 Fig Questionnaire for Allergic Study. (PDF)

S2 Fig HDM Lung Inflammation Model (from [14]). Mice were intranasally exposed to HDM extract as indicated and on day 15 analyzed for AHR, BALF cell distribution, and lung tissue was collected for sectioning.
(PDF)

S1 Table ADAM10 expression in Naïve and Stimulated WT B cells by qPCR. A+I (3 days stimulation with anti-CD40+IL4); A (3 day stimulation with anti-CD40 alone). ADAM10 expression normalized to 18s. Fold change in gene expression of 2 or more between groups considered significant.
(PDF)
