# Supplementary material for: Increased B Cell ADAM10 in Allergic Patients and Th2 Prone Mice
Source: PLoS One. 2015 May 1;10(5):e0124331. doi: 10.1371/journal.pone.0124331 (PMC4416757; doi:10.1371/journal.pone.0124331)
Supplement: S1 Fig — (PDF) [file pone.0124331.s001.pdf]

Questionnaire for Allergic Study
Please answer the questions below to determine your qualification as a control or study subject.

1. A. Have you ever been skin tested for allergies?
A. If yes, were any of the tests positive?

Yes No

B. If yes, what were you allergic to?

____

2. Have you ever been diagnosed by a physician as having allergies to pollen, dust, animals or other common allergens?

Yes No

(If No, stop here)

3. TODAY, do you have any of the follow: (circle Yes or No)

Sneezing	Yes	No
Hives	Yes	No
Itchy/Irritated Skin	Yes	No
Stuffy Nose/Itchy Nose	Yes	No
Itching or Red eyes	Yes	No
Coughing	Yes	No
Wheezing	Yes	No
Difficulty Breathing	Yes	No

4. Have you experienced any of the above symptoms within the last two days? (Circle Yes or No)

Yes No

5. TODAY, are you taking the following medications? (Circle all that apply)
   A. antihistamines
   B. nasal corticosteroids
   C. eye drops
   D. other (please list any other medications that you are currently taking)

____

6. Is today your second blood draw for this study? (Circle Yes or No)

Yes No

If Yes, what was your previous participant number (indicated on the top of your "Instructions for Returning Participants" form)______________________________

Approved
RG/EP 2/20/14

(If No, continue to question 7)

7. Would you be willing to return for a second blood draw when you are no longer symptomatic for at least one month? (Circle Yes or No)

Yes No

If, Yes, please remove the next page. This page includes your study participant number and the contact information for the clinic.

Approved
2/20/14 / R6 / EP
