# Supplementary figures and images for: Increased B Cell ADAM10 in Allergic Patients and Th2 Prone Mice
Source: PLoS One. 2015 May 1;10(5):e0124331. doi: 10.1371/journal.pone.0124331 (PMC4416757; doi:10.1371/journal.pone.0124331)

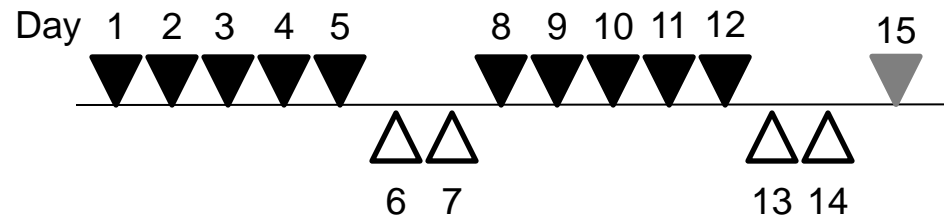

▼ I.N. 15 $\mu$ g/25 $\mu$ L HDM or saline

△ Rest

▼ AHR/BALF/PB/Lung Collection

Supplement: S2 Fig — Mice were intranasally exposed to HDM extract as indicated and on day 15 analyzed for AHR, BALF cell distribution, and lung tissue was collected for sectioning. (PDF) [file pone.0124331.s002.pdf]
