# Supplementary material for: Increased B Cell ADAM10 in Allergic Patients and Th2 Prone Mice
Source: PLoS One. 2015 May 1;10(5):e0124331. doi: 10.1371/journal.pone.0124331 (PMC4416757; doi:10.1371/journal.pone.0124331)
Supplement: S1 Table — A+I (3 days stimulation with anti-CD40+IL4); A (3 day stimulation with anti-CD40 alone). ADAM10 expression normalized to 18s. Fold change in gene expression of 2 or more between groups considered significant. (PDF) [file pone.0124331.s003.pdf]

Comparison	Average Fold Change in ADAM10 expression (±SD) using ΔΔCt
**Balb naïve / C57 naïve**	**2.35 ± 1.00**
**Balb A+I / Balb naïve**	**2.02 ± 0.66**
**C57 A+I / C57 naïve**	**2.40 ± 1.34**
**Balb A / Balb naïve**	**2.88 ± 1.45**
**C57 A / C57 naïve**	**1.91 ± 0.30**
**Balb A+I / C57 A+I**	**5.34 ± 1.84**
**Balb A / C57 A**	**5.70 ± 1.45**
